# Factors That Impact the Relationship between Perceived Organizational Support and Technostress in Teachers

**DOI:** 10.3390/bs13050364

**Published:** 2023-04-27

**Authors:** Patricia Solís, Rocío Lago-Urbano, Sara Real Castelao

**Affiliations:** 1 Faculty of Education, Universidad Internacional de La Rioja, 26006 Logroño, Spain; 2 Psychology Department, University of Huelva, 21004 Huelva, Spain; rocio.lago@dpee.uhu.es; 3 Centro de Ponferrada, Universidad Nacional de Educación a Distancia, 24400 Ponferrada, Spain; sarreal@ponferrada.uned.es

**Keywords:** perceived organizational support, technostress, teachers, technologies, stress

## Abstract

In the last two years, the obligatory use of technologies due to the COVID-19 pandemic has increased the technostress suffered by education professionals. This study investigates the relationships between technostress and perceived organizational support and the influence of certain socio-demographic variables. An online survey was administered to 771 teachers working in different educational stages in various autonomous communities in Spain. Perceived organizational support was found to be significantly correlated with technostress. Women tend to experience more technostress in general and significant gender differences were also found in the dimension of anxiety. The analyzed data also suggest that perceived organizational support is higher in private schools. In urban centers, teachers’ technostress increases in higher educational stages, such as secondary education and baccalaureate. Further work is needed to develop school policies that address the needs of teachers and provide support for those at risk of technostress. In addition, there is a need to design coping strategies and prioritize the most at-risk sectors to improve their overall health and well-being.

## 1. Introduction

Today, stress is regarded as a hidden epidemic [1,2] that has serious negative consequences for employees’ physical and emotional health [3]. Likewise, information and communication technologies (ICT) add to existing work-related stress [4], giving rise to technostress, a problem experienced by individuals who cannot adapt or get used to ICTs [5]. With the effect of the COVID-19 health epidemic, the use of technology has increased, and digital technologies have been used in all fields of life [6], including education and training at all stages and in all areas.

The concept of technostress is multidimensional and was first defined in the 1980s by Brod [7] (p. 16) as “a modern adaptive illness caused by the inability to cope with new technologies in a healthy way”. This construct is now defined as a negative psychological state associated with the “threat” of using new technologies,” leading to “anxiety, mental fatigue, skepticism and a sense of inefficacy” [8] and has been studied in-depth to determine its causes and negative effects [9,10].

The current technological developments and advancements in work tasks highlight the importance for companies of considering the potential impact of technostress on employees [11]. Thus, it has been studied how and why ICT use causes people to respond to the associated demands in a way that is stressful for them [12]. This issue has been studied in different work sectors, including business professionals [13], public administrators [13], academics [14], teachers [15,16] and university students [17].

On the other hand, according to organizational support theory, employees generally have a perception of how their work organization values their contribution and cares about their well-being [18]. This perceived organizational support positively affects job and life satisfaction [19], thus supporting the claim that companies should pay attention to the technostress of their employees and provide them with appropriate coping strategies.

Technostress also affects teachers, for whom perceived organizational support is also vital. Teachers, particularly those working at compulsory levels, are leaving the profession at an alarming rate, and staff turnover is high in many schools [20], this being one of the professions at risk of high levels of stress and syndromes such as burnout [21]. Thus, we must focus on the causes of these situations, including lack of support, poor leadership, low pay, and low job satisfaction [22].

## 2. Theoretical Background

### 2.1. Teachers’ Technostress

Today, society’s immediate need to digitize has accelerated ICT implementation in education [23,24]. Digital tools undoubtedly benefit teaching, but they are also linked to negative factors such as fatigue and stress [25]. Teachers, particularly those teaching at basic stages, suffer from high levels of stress that worryingly impact their quality of life [26]. Numerous research studies have focused on the harms generated by technostress and how it affects productivity and health [13,27], job satisfaction, the intention to use technology [28], and organizational commitment [29]. However, there is a lack of empirical studies aimed at developing strategies for appropriately managing ICT-induced stress [30].

Several external factors influence teachers’ efforts to integrate ICT into their methodologies and manifest themselves as technostress, including business management, educational policies, communication and collaboration with colleagues, and particularly being unrewarded or unrecognized for their efforts or being unable to meet expectations [31]. Five main causes of teacher technostress [32] have emerged from the literature: individual problems (self-efficacy, attitude, and financial situation), technical problems, education-oriented problems, health problems, and time problems. Similarly, ICT competence, alignment of educational ICT use with teaching style, school support, and attitudes towards educational ICT use are key predictors of technostress [24]. Unsurprisingly, self-perceived ICT competence reduces teachers’ technostress [16].

To understand the presence and nature of technostress, numerous researchers have designed specific scales or instruments. There are different tools for assessing technostress, such as checklists, observation, interviews, and self-report questionnaires. The self-report questionnaire, due to its practical utility and ease of use, is the most commonly used tool for assessing technostress. Notable in this field is the Technostress Experience Evaluation Questionnaire, developed by Tarafdar et al. [33]. These authors developed and validated a diagnostic tool to determine the extent to which technostress is present in an organization. Later, these authors published a study considered a milestone in this field [34] in which they developed a measurement scale that has been one of the most widely used in the literature. Parallelly in the Spanish context, the team of researchers led by Salanova has extensively studied the technostress phenomenon, but they have gone one step further by focusing on how to measure it. For this reason, they have created different instruments with a common theoretical framework that, based on the scientific results obtained and their use, have been improved (Questionnaire of Experiences Related to Work, Questionnaire of Resources, Emotions and Demands, General Resources, Emotions/Experiences and Demands Questionnaire) [9]. In this way, they have managed to build the evaluation tool that we currently have (RED-TIC Technostress Questionnaire) and its different versions for specific samples. It is a valid and reliable evaluation tool for studying the phenomenon in the Spanish context.

A deeper understanding of teacher technostress therefore requires a closer look at the organization’s environmental factors along with internal and individual factors [10].

### 2.2. Perceived Organizational Support

Perceived organizational support is related to technostress as it can moderate its impact [35]. The actions of educational management teams generate working conditions that can positively (or negatively) influence teachers’ motivation, well-being, or professional practice [36]. If organizations treat their employees with care and pay attention to them, this will convey the message that the organization values them [37]. Thus, teachers will pursue educational innovation when they feel that the school supports their competence and autonomy, that is, when there is perceived organizational support [38,39], which is a key element in teachers’ job satisfaction. In addition to the perceived support from their organizations, the availability of resources and technical support for incidents are also important for teachers to implement ICT in their teaching [40].

Evaluating perceived organizational support in teachers is important for several reasons. It allows educators to identify areas where support may be lacking, and to develop strategies to improve it. Additionally, it can help to promote job satisfaction, well-being, and retention among teachers, ultimately leading to better educational outcomes for students. One of the first instruments used to evaluate this area was the Survey of Perceived Organizational Support, developed by Eisenberger, Huntington, Hutchison, and Sowa (1986) [41]. The Spanish version of the Work Environment Scale (WES) [42] has also been extensively used. However, the Eisenberger questionnaire has a longer trajectory and was created with the aim of evaluating workers’ beliefs about the support provided by the companies where they carry out their work activities.

### 2.3. Variables Influencing the Level of Technostress and Perceived Organizational Support

#### 2.3.1. Gender

Gender is often suggested as one of the variables to be considered when studying technostress [9,10,43]. Some studies have found significant differences in technostress according to gender, with women showing a greater tendency to perceive technostress [17,44,45]. However, others report the opposite, with technostress scores being higher for men [28,46,47]. Finally, some studies have found no significant gender differences [48,49]. Technostress in Spanish teachers is a phenomenon that has been studied in scientific research. A study found that excessive use of information and ICT can increase the level of stress in teachers and negatively affect their physical and emotional health [35]. Other studies find differences according to gender, with women experiencing greater difficulties in balancing their professional and personal lives [50,51]. 

#### 2.3.2. Type, Scope, and Stage of the Educational Establishment

There is a lack of research that explores how technostress could be affected by the type of school (public or private) and its setting (urban or rural). However, Chou and Chou report that teachers in public schools are less willing to continue teaching online after the pandemic than teachers in public schools [52]. Similarly, Marawan et al. found higher levels of technostress among teachers in rural settings [53].

Several studies have compared general teaching stress between those working in primary and secondary stages. However, these studies have yielded mixed results and no firm conclusions can be drawn. While some studies indicate that teachers working with secondary students present greater levels of stress [16,54,55], others report that the prevalence of stress is higher for those who work in primary education [21,56].

#### 2.3.3. Type of Contractual Relationship with the Center 

The contractual relationship between teachers and the educational center (and therefore their stability within the organization) concerning organizational support and technostress, has been analyzed in various studies [26]. From the standpoint of organizations representing workers in the education sector [57], contractual stability is considered one of the risk factors for the profession, due to the problems associated with relocation (including personal and family issues). Other factors such as working conditions in recent years, such as job insecurity, personal fulfillment, longer teaching hours, lower salary, etc., have also been studied, finding that these can lead to higher levels of anxiety and burnout among younger professionals in Spain [58].

### 2.4. Research Model and Hypotheses

**Figure 1:**
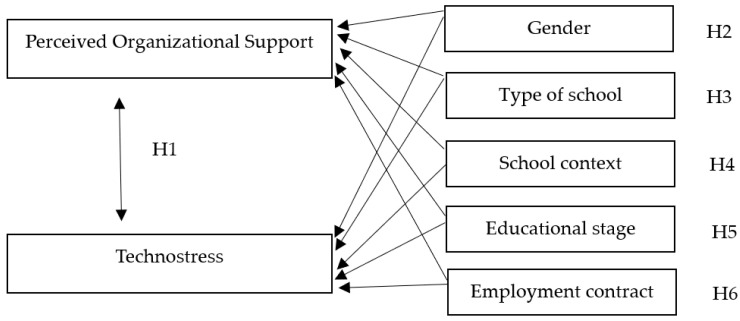
Hypothesized research model.

The relationships between technostress and perceived organizational support and, on the other hand, the influence of selected socio-demographic variables are of interest in this study and shape the research hypotheses illustrated in the research model in Figure 1.

- **H1:** *There is a relationship between technostress and perceived organizational support*.
- **H2:** *Gender influences technostress and also the perceived organizational support levels of teachers*.
- **H3:** *Type of school influences technostress and also the perceived organizational support levels of teachers*.
- **H4:** *School Context influences technostress and also the perceived organizational support levels of teachers*.
- **H5:** *Educational Stage influences technostress and also the perceived organizational support levels of teachers*.
- **H6:** *Type of employment contract influences technostress and also the perceived organizational support levels of teachers*.

## 3. Materials and Methods

### 3.1. Participants

The sample consisted of 771 teachers (565 women and 206 men) from 18 autonomous communities in Spain, the most represented being Castilla y León (18%), Catalonia (13%), Castilla La Mancha (12%), and Navarra (10%). In the sample, 65% of the educational centers were in urban areas and 35% in rural areas. Of the teachers in the sample, 80% work in public schools and 20% work in private or state-subsidized schools. Furthermore, 68% of the participants have a civil servant or permanent contract, while 32% are interim or have a temporary contract.

The mean age of the sample is 44.12 years (SD = 9.71) (women: 43.87 years; men: 44.82), with a range between 23 and 67 years. The average length of teaching experience is 15.98 years (SD = 10.30) (15.76 for women and 16.60 for men).

Concerning the training of the teachers in the sample, 60% have a bachelor’s degree (of these, 26% have completed the Pedagogical Accreditation Course, and 17% have completed the master’s degree in teacher training). In addition, 28% have completed primary education (24% have obtained a diploma and 4% a degree), 8% have studied early childhood education (7% have completed a diploma and 1% a degree). Finally, 4% have also studied for a doctorate. 

Concerning the stages in which they carry out their teaching work, 34% do so in primary education, 32% in baccalaureate and vocational training, 24% in compulsory secondary education, and 10% in early childhood education.

### 3.2. Procedure

First, the authors contacted the management teams of several schools in Spain to provide them with information on the content and objectives of this study. Subsequently, those schools interested in participating were provided with a digital link to complete the self-reports. In compliance with the requirements of the Research Ethics Committee, anonymous and voluntary completion was ensured without any reward. Furthermore, to avoid potential bias in the responses, there was a commitment to not analyzing the participants’ answers individually.

### 3.3. Variables and Measurement Instruments

The variables studied are the following:

- Variable “x”: Perceived Organizational Support, refers to the extent to which employees believe that their organization values their contributions and cares about their well-being. It is measured through employee perceptions.
- Variable “y”: Technostress, refers to the negative psychological and emotional reactions that people experience as a result of their interactions with technology.
- Variable “a”: Gender, dichotomized as male and female.
- Variable “b”: Type of School, dichotomized as public and private-subsidized schools.
- Variable “c”: School Context, dichotomized as urban and rural
- Variable “d”: Educational Stage, categorized as primary education, compulsory secondary education and baccalaureate/vocational training
- Variable “e”: Employment Contract, dichotomized as permanent contract and temporary contract.

The 771 teachers who agreed to participate completed two standardized evaluation instruments:

- Survey of Perceived Organizational Support [53]. This instrument evaluates workers’ perceptions of the support they receive from the organization to which they belong. It is based on the theory of social exchange, which holds that employees are motivated to contribute to their organization’s success when they feel that the organization treats them fairly and offers them adequate support. The questionnaire has a unidimensional short form of 17 items that measure the perception of support that employees receive from their organization in different areas, such as emotional support, instrumental support, and recognition, with seven-point Likert-type responses ranging from “strongly disagree” to “strongly agree.” The scores from the items are summed to obtain a total score of perceived support by the employee. The SPOS has been used in numerous research studies in different types of organizations, and it has been found to have good validity and reliability. It has a Cronbach’s alpha of 0.93. This short version was validated in Spanish [54], obtaining a Cronbach’s Alpha of 0.78. It has already been applied in several studies [55,56,57]. The survey results can help managers and leaders of an organization to better understand how organizational support is perceived by employees, and to identify areas where support and motivation of the staff can be improved.
- RED-TIC Technostress Questionnaire [8]. This instrument is a questionnaire designed to measure the level of technostress experienced by workers due to the use of information and communication technologies (ICT) in the workplace. It distinguishes four variables related to the use of technologies at work (managing data, ICT use, psychosocial risks, and psychosocial consequences) and consists of four scales: fatigue, anxiety, skepticism, and ineffectiveness. The items are rated on a five-point Likert scale, from “never” to “always”. The scores from the items are summed to obtain a total score of technostress experienced by the worker. It has obtained alpha values above 0.83 in all dimensions and has been used in previous studies [42,46]. The survey results can help managers and leaders of the organization to better understand how ICT affects the well-being of workers and to identify areas where technostress can be reduced and the health and productivity of employees can be improved.

The authors used the statistical package SPSS 25.0 for Windows for data analysis.

## 4. Results

We first describe the total scores for the variables Perceived Organizational Support, Technostress, and its four dimensions (Inefficacy, Skepticism, Fatigue, and Anxiety). From a possible score ranging from 1–7, participants obtained a mean score on the Perceived Organizational Support variable of 4.74 (SD = 1.28). The mean score for Technostress (from a possible score of 0–7) was 1.87 (SD = 1.34), with the dimensions distributed as follows: ineffectiveness (x = 1.48, SD = 1.36, range 0–6), skepticism (x = 1.72, SD = 1.63, range 0–6), fatigue (x = 2.43, SD = 1.91, range 0–6) and anxiety (x = 1.88, SD = 1.68, range 0–6).

### 4.1. Correlation between Technostress and Perceived Organizational Support

Non-parametric correlation analyses were conducted using Spearman’s Rho test. It was found that perceived organizational support correlated significantly (at the 0.05% level) with technostress (r = −0.25) and all its components.

### 4.2. Differences According to Socio-Demographic Variables

The assumption of normality and equality of variances (homoscedasticity) was tested using the Kolmogorov–Smirnov and Shapiro–Will statistics, Q–Q plots, and Levene’s test (based on means). Since the sample does not meet the assumptions of normality, non-parametric tests were subsequently used.

We also used the Mann–Whitney U test to explore possible differences according to gender (male and female), type of school (public and private-subsidized), type of contract (permanent and temporary), and school setting (rural and urban) in perceived organizational support and technostress.

#### 4.2.1. Gender

As shown in Table 1, women obtained higher average scores than men on all the dimensions except for Perceived Organizational Support (men scored 13.97 points higher). These differences were significant for the anxiety dimension (*p* < 0.05), although in the Technostress variable, the value of the statistic is very close to chance level (0.05).

#### 4.2.2. Type of School

Regarding the type of school (Table 2), teachers at public schools show higher mean scores for the variable technostress and all its dimensions, while Perceived Organizational Support is significantly higher for those working in private-subsidized schools.

#### 4.2.3. School Context

Concerning the school setting (Table 3), the average scores are higher for Perceived Organizational Support and Technostress for teachers in urban schools. However, in the case of the Skepticism dimension, the mean is higher for teachers in rural schools, although the difference is non-significant.

#### 4.2.4. Educational Stage

No significant differences were found between infant and primary or between primary and secondary school teachers on any of the study variables. However, significant differences were found between teachers in compulsory secondary education and those who teach baccalaureate and vocational training (Table 4), with higher mean scores for Technostress and significant differences in the four dimensions that comprise the scale (Skepticism, Fatigue, Anxiety, and Inefficacy).

#### 4.2.5. Employment Contract

Teachers with permanent contracts obtained higher scores on Perceived Organizational Support and Technostress. However, in the Fatigue sub-dimension belonging to Technostress, the scores were higher for those teachers with a temporary contract. None of the differences were statistically significant.

## 5. Discussion

While the use of technology offers flexibility and simplifies certain processes, it has also exacerbated the problem of technological stress. Furthermore, the use of ICT can sometimes pose a threat due to its misuse or overuse, resulting in technostress—something that has been more evident in the last ten years, presenting itself as an occupational risk challenge [23,59]. Moreover, as discussed in the theoretical framework, technostress is related to perceived organizational support. This contributes to job satisfaction by conveying to employees that help and support are always available [20]. Therefore, this study proposed six hypotheses for analyzing both the relationship between technostress and perceived organizational support in teachers (Hypothesis 1) and the influence of certain socio-demographic variables on this relationship (Hypotheses 2 to 6: gender, type, and setting of school, educational stage, and type of employment contract).

First, moderate scores were found for perceived organizational support and technostress, in accord with other studies [6]. Emphasis should be placed on the former variable as teachers who receive support from their school are happier and tend to be more self-fulfilled [60]. This should be a goal of education systems in the 21st century.

Regarding the relationship between perceived organizational support and technostress, the correlation between the two variables is consistent with previous studies examining the relationship between perceived employer support and employee stress [33,61].

Concerning socio-demographic variables, the results of this study indicate that women tend to experience more technostress in general, and significant gender differences were found in the anxiety dimension. These results are consistent with other studies on teachers [17,62]. Furthermore, La Torre also confirmed that techno overload, technoinvasiveness, and tech complexity—which are related to technostress—were significantly associated with the female gender [45]. Similar results were found by Marchiori, as women reported being subject to higher levels of tech complexity and techno-uncertainty [43].

Although higher levels of technostress were found in public schools, the differences were not significant. However, teachers from private schools indicated significantly higher levels of perceived organizational support than those working in public schools. It should be recalled that school administrators are expected to address teachers’ needs and provide technical assistance and educational resources [52].

Regarding the school setting, teachers in urban centers obtained higher scores on both Perceived Organizational Support and Technostress. Estrada-Muñoz et al. point out that working in schools, especially in urban areas, is related to greater stress and burnout in teachers [46]. 

Concerning the educational stage, significant differences were only found for the variable technostress. That is, teachers in compulsory secondary education obtained higher scores on the four dimensions than those who teach on baccalaureate and vocational training programs. These findings are in agreement with general data reported on technostress in secondary schools [16], although no previous studies have analyzed the difference between compulsory and baccalaureate levels. 

Although the type of employment contract has barely been studied in the available scientific literature concerning organizational support and technostress, it might be assumed that temporary contracts are linked to greater technostress and less perceived organizational support due to the precariousness and uncertainty of the employment situation. Contrary to these expectations, teachers with permanent contracts showed higher average levels of Perceived Organizational Support and Technostress than those with temporary contracts (although these differences were non-significant). However, in the Fatigue dimension belonging to the Technostress variable, the scores were higher for teachers with a temporary contract. One explanation for this finding could be that teachers with low motivation to teach may regard their work only as a paid activity and even a burden [20].

## 6. Conclusions

The results of this study suggest that the use of technology in education may increase technological stress, which in turn can affect teachers’ job satisfaction. Furthermore, the results indicate that perceived organizational support for teachers can reduce technological stress and increase their job satisfaction. 

Significant differences were found in the technological stress experienced by teachers based on gender, with women reporting higher levels of technological stress overall. In addition, teachers working in urban environments reported higher levels of perceived organizational support and technological stress than those working in rural environments.

Practical recommendations for educational institutions include promoting organizational support and providing technology skill training to reduce teachers’ technological stress. Gender differences should also be taken into account, and additional resources should be provided for teachers working in urban environments.

Attention should also be paid to the employment status of teachers, as those with temporary contracts reported higher levels of fatigue related to technological stress. Therefore, additional resources and support should be provided for teachers with temporary contracts to help reduce their technological stress.

In summary, the results of this study highlight the importance of organizational support and proper technology management in reducing technological stress in teachers and improving their job satisfaction. Educational institutions can use these results to implement policies and programs that promote a healthy and satisfying work environment for teachers.

Considering the data presented in this study, it is clearly necessary to pursue lines of research that could help to inform the design of coping strategies for dealing with technostress and organizational plans that mitigate the risk factors affecting teachers when using ICT. As already pointed out in other review studies on technostress [31,46], there is a need to target those socio-demographic profiles that are at greater risk of suffering high levels of technostress that affect their job satisfaction and, in the medium and long term, their health and overall well-being.

This study has certain limitations that should be considered. Of note are the possibilities of access to the sample and the type of instruments employed. In particular, we recruited a non-random sample, and the data come from self-report measures, the results of which may be biased by social desirability. However, these limitations are common to this research area in general and the previous studies described here (with which we compare our findings).

The strengths of this study include the size and geographical diversity of the sample, since our participants were recruited from 18 different autonomous communities, and from different educational stages and modalities. This diversity has allowed us to analyze the different variables of gender, type of school, educational stage, and contract type with a large sample size.

## Figures and Tables

**Table 1:** Descriptive data and Mann–Whitney test results for the gender variable.

		*N*	Perceived Organizational Support	Technostress	Skepticism	Fatigue	Anxiety	Inefficacy
Mid-range	Women	565	382.27	399.64	391.61	398.40	401.19	394.46
	Men		396.24	348.59	370.61	351.99	344.34	362.80
Mann–Whitney U test			56,086.50	50,489.50	55,024.00	51,189.50	49,613.50	53,416.000
W for Wilcoxon			215,981.50	71,810.50	76,345.00	72,510.50	70,934.50	74,737.000
Z			−0.771	−2.816	−1.171	−2.567	−3.148	−1.757
Sig. asymptotic. (bilateral)			0.441	0.005 *	0.242	0.01 *	0.002 *	0.079

Notes: (*N* = 771), * for *p* < 0.01; (two-tailed).

**Table 2:** Descriptive data and Mann–Whitney test results for the type of center variable.

		*N*	Perceived Organizational Support	Technostress	Skepticism	Fatigue	Anxiety	Inefficacy
Mid-range	Public	618	350.49	392.89	391.66	395.74	392.59	386.13
	Private-subsidized		529.44	358.17	363.12	346.67	359.40	385.46
Mann–Whitney U test			25,331.00	43,018.50	43,777.00	41,260.000	43,206.500	47,194.000
W for Wilcoxon			216,602.00	54,799.50	55,558.00	53,041.000	54,987.500	58,975.000
Z			−8.899	−1.727	−1.434	−2.446	−1.657	−0.034
Sig. asymptotic. (bilateral)			0.001 **	0.084	−0.152	0.014 *	0.098	0.973

Notes: (*N* = 771), * for *p* < 0.05; ** for *p* < 0.001 (two-tailed).

**Table 3:** Descriptive data and Mann–Whitney test results for the school setting variable.

		*N*	Perceived Organizational Support	Technostress	Skepticism	Fatigue	Anxiety	Inefficacy
Mid-range	Urban	502	397.91	389.23	381.98	392.51	389.28	389.37
	Rural	269	363.77	379.97	393.51	373.84	379.88	379.72
Mann–Whitney U test			61,540.00	65,897.00	65,498.50	64,248.50	65,872.00	65,829.00
W for Wilcoxon			97,855.00	102,212.00	191,751.500	100,563.50	102,187.00	102,144.00
Z			−2.029	−0.550	0.693	−1.113	0.561	−0.577
Sig. asymptotic. (bilateral)			0.042 *	0.582	0.489	0.266	0.575	0.564

Notes: (*N* = 771), * for *p* < 0.05. (two-tailed).

**Table 4:** Descriptive data and Mann–Whitney test results for the educational stage variable.

		*N*	Perceived Organizational Support	Technostress	Skepticism	Fatigue	Anxiety	Inefficacy
Mid-range	Compulsory Secondary Education	183	229.55	202.40	213.92	200.03	201.53	202.79
	Baccalaureate/Vocational Training		207.82	227.69	219.25	229.42	228.32	227.40
Mann–Whitney U test			20,579.00	63,115.50	60,789.50	63,780.00	63,833.50	61,713.50
W for Wilcoxon			51,954.00	94,490.50	92,164.50	199,761.00	95,208.50	93,088.50
Z			−1.78	−0.44	−2.42	−2.21	−2.034	−2.077
Sig. asymptotic. (bilateral)			0.07	0.65	0.016 *	0.027 *	0.042 *	0.038 *

Notes: (*N* = 771), * for *p* < 0.05; (two-tailed).

## Data Availability

The data presented in this study are available on request from the corresponding author. The data are not publicly available due to privacy considerations.

## References

[1] Jacobs C.M. (2019). Ineffective-leader-induced occupational stress. SAGE Open.

[2] Peterson C.L., Peterson C.L., Mayhew C. (2018). The epidemic of stress. Occupational Health and Safety.

[3] Gonçalves A., Fontes L., Simães C., Gomes A.R., Arezes P., Baptista J., Barroso M., Carneiro P., Cordeiro P., Costa N., Melo R., Miguel A., Perestrelo G. (2019). Stress and burnout in health professionals. Occupational and Environmental Safety and Health.

[4] Atanasoff L., Venable M.A. (2017). Technostress: Implications for adults in the workforce. Career Dev. Q..

[5] Nimrod G. (2018). Technostress: Measuring a new threat to well-being in later life. Aging Ment. Health.

[6] Çoklar A.N., Bozyiğit R. (2021). Determination of Technology Attitudes and Technostress Levels of Geography Teacher Candidates. Int. J. Geogr. Geol. Educ..

[7] Brod C. (1984). Technostress: The Human Cost of the Computer Revolution.

[8] Salanova M., Llorens S., Cifre E., Nogareda C. (2007). El Tecnoestrés: Concepto, Medida e Intervención Psicosocial.

[9] Llorens S., Salanova M., Ventura M. (2011). Tecnoestrés. Intervention Guides.

[10] Özgür H. (2020). Relationships between teachers’ technostress, technological pedagogical content knowledge (TPACK), school support and demographic variables: A structural equation modeling. Comput. Hum. Behav..

[11] Tarafdar M., Cooper C.L., Stich J.F. (2019). The technostress trifecta-techno eustress, techno distress and design: Theoretical directions and an agenda for research. Inf. Syst. J..

[12] Tarafdar M., Pullins E.B., Ragu-Nathan T.S. (2015). Technostress: Negative effect on performance and possible mitigations. Inf. Syst. J..

[13] Fuglseth A.M., Sørebø Ø. (2014). The effects of technostress within the context of employee use of ICT. Comput. Hum. Behav..

[14] Jena R. (2015). Technostress in ICT enabled collaborative learning environment: An empirical study among Indian academician. Comput. Hum. Behav..

[15] Ansley B.M., Houchins D.E., Varjas K., Roach A., Patterson D., Hendrick R. (2021). The impact of an online stress intervention on burnout and teacher efficacy. Teach. Teach. Educ..

[16] Joo Y.J., Lim K.Y., Kim N.H. (2016). The effects of secondary teachers’ technostress on the intention to use technology in South Korea. Comput. Educ..

[17] Wang X., Tan S.C., Li L. (2020). Technostress in university students’ technology-enhanced learning: An investigation from multidimensional person-environment misfit. Comput. Hum. Behav..

[18] Eisenberger R., Rhoades Shanock L., Wen X. (2020). Perceived organizational support: Why caring about employees counts. Annu. Rev. Organ. Psychol..

[19] Bachtiar D., Sudibjo N., Bernarto I. (2018). The effects of transformational leadership, perceived organizational support on job and life satisfaction of preschool teachers. Int. Inf. Inst..

[20] Bernarto I., Bachtiar D., Sudibjo N., Suryawan I.N., Purwanto A., Asbari M. (2020). Effect of transformational leadership, perceived organizational support, job satisfaction toward life satisfaction: Evidences from Indonesian teachers. Int. J. Adv. Sci. Technol..

[21] Extremera N., Rey L., Pena M. (2010). Teaching seriously damages health. Analysis of symptoms associated with teaching stress. Boletín Psicol..

[22] Chambers R. (2010). Paradigms, poverty and adaptive pluralism. IDS Work. Pap..

[23] Jurek P., Korjonen-Kuusipuro K., Olech M. (2021). When technology use causes stress: Challenges for contemporary research. Hum. Technol..

[24] Syvänen A., Mäkiniemi J.P., Syrjä S., Heikkilä-Tammi K., Viteli J. (2016). When does the educational use of ICT become a source of technostress for Finnish teachers?. Seminar.

[25] González S.B.G., Pérez S.F. (2019). Tecnoestrés docente: El lado opuesto de la utilización de las nuevas tecnologías por los Docentes del Nivel Medio. Rev. Cient. Estud. Investig..

[26] Idoiaga N., Berasategi N., Santamaria M.D., Ozamiz-Etxebarria N. (2021). Reopening of Schools in the COVID-19 Pandemic: The Quality of Life of Teachers While Coping with This New Challenge in the North of Spain. Int. J. Environ. Res. Public Health.

[27] Tarafdar M., Tu Q., Ragu-Nathan T.S., Ragu-Nathan B.S. (2011). Crossing to the dark side: Examining creators, outcomes, and inhibitors of technostress. Commun. ACM.

[28] Maier C., Laumer S., Eckhardt A. (2015). Information technology as daily stressor: Pinning down the causes of burnout. J. Bus. Econ..

[29] Ungku U.A., Amin Salmiah M.A., Wan W.I. (2014). Moderating effect of technostress inhibitors on the relationship between technostress creators and organisational commitment. J. Teknol.-Sci. Eng..

[30] Cuervo T., Orviz N., Arce S., Fernández I.S. (2018). Technostress in the Society of Technology and Communication: A Bibliographic Review from the Web of Science. Arch. Prev. Riesgos Labor..

[31] Voet M., De Wever B. (2017). Towards a differentiated and domain-specific view of educational technology: An exploratory study of history teachers’ technology use. Br. J. Educ. Technol..

[32] Çoklar A., Efilti E., Şahin Y., Akçay A. (2016). Determining the reasons for technostress experienced by teachers: A qualitative study. Turk. Online J. Qual. Inq..

[33] Tarafdar M., Tu Q., Ragu-Nathan B.S., Ragu-Nathan T.S. (2007). The impact of technostress on role stress and productivity. J. Manag. Inf. Syst..

[34] Ragu-Nathan T.S., Tarafdar M., Ragu-Nathan B.S., Tu Q. (2008). The consequences of technostress for end users in organizations: Conceptual development and empirical validation. Inf. Syst. Res..

[35] Xu Z., Yang F. (2018). The impact of perceived organizational support on the relationship between job stress and burnout: A mediating or moderating role?. Curr. Psychol..

[36] Ford T.G., Olsen J., Khojasteh J., Ware J., Urick A. (2019). The effects of leader support for teacher psychological needs on teacher burnout, commitment, and intent to leave. J. Educ. Adm..

[37] Shahi S., Andarz S., Andarz K., Yasini M. (2017). The relationship between organizational justice and perceived organizational support with the desire to leave the occupation knowledge workers. Organ. Resour. Manag. Res..

[38] Sadaf A., Johnson B.L. (2017). Teachers’ beliefs about integrating digital literacy into classroom practice: An investigation based on the theory of planned behavior. J. Digit. Learn. Teach. Educ..

[39] Scherer R., Howard S.K., Tondeur J., Siddiq F. (2021). Profiling teachers’ readiness for online teaching and learning in higher education: Who’s ready?. Comput. Hum. Behav..

[40] Khlaif Z. (2018). Teacher’ perceptions of factors affecting their adoption and acceptance of mobile technology in K-12 settings. Comput. Sch..

[41] Eisenberger R., Huntington R., Hutchison S., Sowa D. (1986). Perceived organizational support. J. Appl. Psychol..

[42] Moos R. (2008). A Social Climate Scale, Work Environment Scale Manual, Development, Applications, Research.

[43] Marchiori D.M., Mainardes E.W., Rodrigues R.G. (2019). Do individual characteristics influence the types of technostress reported by workers?. Int. J. Hum.-Comput. Interact..

[44] La Torre G., De Leonardis V., Chiappetta M. (2020). Technostress: How does it affect the productivity and life of an individual? Results of an observational study. Public Health.

[45] Rey-Merchán M.C., López-Arquillos A., Sánchez E., Colomo E., Palmero J.R., Coords J.S. (2020). Prevention and management of technostress as an occupational risk among the teaching profession. Tecnologías Educativas y Estrategias Didácticas.

[46] Estrada-Muñoz C., Vega-Muñoz A., Castillo D., Müller-Pérez S., Boada-Grau J. (2021). Technostress of Chilean Teachers in the Context of the COVID-19 Pandemic and Teleworking. Int. J. Environ. Res. Public Health.

[47] Jena R.K., Mahanti P.K. (2014). An empirical study of Technostress among Indian academicians. Int. J. Educ. Learn..

[48] Hsiao K.L., Shu Y., Huang T.C. (2017). Exploring the effect of compulsive social app usage on technostress and academic performance: Perspectives from personality traits. Telemat. Inform..

[49] Prendes-Espinosa M.P., García-Tudela P.A., Solano-Fernández I.M. (2020). Igualdad de género y TIC en contextos educativos formales: Una revisión sistemática. Comunicar.

[50] Picón C., Toledo S., Navarro V. (2017). Tecnoestrés: Identificación y prevalencia en el personal docente de la Facultad de Medicina de la Universidad Nacional del Nordeste. Revista de la Facultad de Medicina.

[51] Solís P., Lago R., Real S. (2021). Consequences of COVID-19 Confinement for Teachers: Family-Work Interactions, Technostress, and Perceived Organizational Support. Int. J. Environ. Res. Public Health.

[52] Chou H.L., Chou C. (2021). A multigroup analysis of factors underlying teachers’ technostress and their continuance intention toward online teaching. Comput. Educ..

[53] Marawan H., Soliman S., Allam H.K., Raouf S.A. (2021). Technostress and remote Virtual work environment among University Staff Members: A cross-sectional study. Environ. Sci. Pollut. Res. Int..

[54] Domenech F. (2009). Self-efficacy, school resources, job stressors and burnout in Spanish primary and secondary school teachers: A structural equation approach. Educ. Psychol..

[55] Guerrero-Barona E., Gómez del Amo R., Moreno-Manso J.M., Guerrero-Molina M. (2018). Psychosocial risk factors, perceived stress and mental health in teachers. Rev. Clin. Contemp..

[56] Ozamiz-Etxebarria N., Berasategi Santxo N., Idoiaga Mondragon N., Dosil Santamaría M. (2020). The Psychological State of Teachers During the COVID-19 Crisis: The Challenge of Returning to Face-to-Face Teaching. Front. Psychol..

[57] FETE-UGT (2009). Stress in the Secondary Education Sector.

[58] Avargues M., Borda M., López A. (2010). El core of burnout y los síntomas de estrés en el personal de Universidad. Prevalencia e influencia de variables de carácter sociodemográfico y laboral. Boletín Psicol..

[59] Brivio E., Gaudioso F., Vergine I., Mirizzi C.R., Reina C., Stellari A., Galimberti C. (2018). Preventing Technostress Through Positive Technology. Front. Psychol..

[60] Cullen-Lester K., Edwards B.D., Casper W.C., Gue K. (2014). Employees’ adaptability and perceptions of change-related uncertainty: Implications for perceived organizational support, job satisfaction and performance. J. Bus. Psychol..

[61] Ahraemi K., Mor M. (2015). The mediating roles of leader-member exchange and perceived organizational support in the role stress-turnover intention relationship among child welfare workers: A longitudinal analysis. Child. Youth Serv. Rev..

[62] Li L., Wang X. (2020). Technostress inhibitors and creators and their impacts on university teachers’ work performance in higher education. Cog. Tech. Work.

